# 4-Formyl-2-nitro­phenyl 4-bromo­benzoate

**DOI:** 10.1107/S1600536813010830

**Published:** 2013-04-27

**Authors:** Rodolfo Moreno-Fuquen, Geraldine Hernandez, Javier Ellena, Carlos A. De Simone, Juan C. Tenorio

**Affiliations:** a Departamento de Química – Facultad de Ciencias, Universidad del Valle, Apartado 25360, Santiago de Cali, Colombia; b Instituto de Física de São Carlos, IFSC, Universidade de São Paulo, USP, São Carlos, SP, Brazil

## Abstract

In the title compound, C_14_H_8_BrNO_5_, the benzene rings form a dihedral angle of 62.90 (7)°. The central ester group is twisted away from the nitro-substituted and bromo-substituted rings by 71.67 (7) and 8.78 (15)°, respectively. The nitro group forms a dihedral angle of 7.77 (16)° with the benzene ring to which it is attached. In the crystal, mol­ecules are linked by weak C—H⋯O inter­actions, forming *C*(12) chains which run along [001]. Halogen–halogen inter­actions [Br⋯Br = 3.523 (3) Å] within the chains stabilized by C—H⋯O inter­actions are observed.

## Related literature

For medicinal and pharmaceutical properties of nitro­aromatic compounds, see: Jefford & Zaslona (1985 ▶); Bhattacharya *et al.* (2006 ▶); Benedini *et al.* (1995 ▶); For similar structures, see: Moreno-Fuquen *et al.* (2011 ▶, 2013 ▶); Moreno-Fuquen (2011 ▶). For van der Waals radii, see: Bondi (1964 ▶). For halogen–halogen inter­actions see Awwadi *et al.* (2006 ▶); Hathwar *et al.* (2010 ▶). For hydrogen bonding, see: Etter (1990 ▶); Nardelli (1995 ▶).
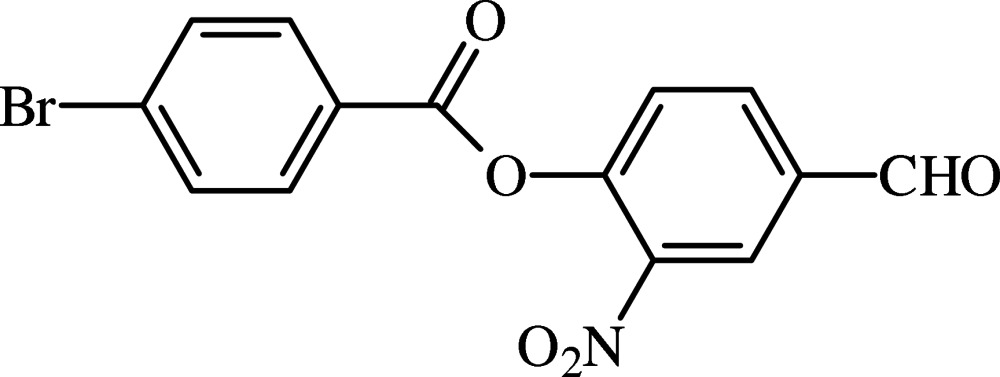


## Experimental

### 

#### Crystal data

C_14_H_8_BrNO_5_
*M* *_r_* = 350.12
Monoclinic, 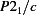

*a* = 6.5308 (2) Å
*b* = 8.2253 (2) Å
*c* = 25.6860 (8) Å
β = 103.1910 (9)°
*V* = 1343.38 (7) Å^3^
*Z* = 4
Mo *K*α radiation
μ = 3.08 mm^−1^
*T* = 295 K
0.38 × 0.16 × 0.12 mm

#### Data collection

Nonius KappaCCD diffractometer
Absorption correction: multi-scan (*SADABS*; Sheldrick, 1996 ▶) *T*_min_ = 0.568, *T*_max_ = 0.687
16446 measured reflections
2746 independent reflections
2095 reflections with *I* > 2σ(*I*)
*R*_int_ = 0.086

#### Refinement

*R*[*F* ^2^ > 2σ(*F* ^2^)] = 0.041
*wR*(*F* ^2^) = 0.113
*S* = 1.03
2746 reflections
190 parameters
H-atom parameters constrained
Δρ_max_ = 0.43 e Å^−3^
Δρ_min_ = −0.39 e Å^−3^

### 

Data collection: *COLLECT* (Nonius, 2000 ▶); cell refinement: *SCALEPACK* (Otwinowski & Minor, 1997 ▶); data reduction: *DENZO* (Otwinowski & Minor, 1997 ▶) and *SCALEPACK*; program(s) used to solve structure: *SHELXS97* (Sheldrick, 2008 ▶); program(s) used to refine structure: *SHELXL97* (Sheldrick, 2008 ▶); molecular graphics: *ORTEP-3 for Windows* (Farrugia, 2012 ▶) and *Mercury* (Macrae *et al.*, 2006 ▶); software used to prepare material for publication: *WinGX* (Farrugia, 2012 ▶).

## Supplementary Material

Crystal structure: contains datablock(s) I, global. DOI: 10.1107/S1600536813010830/hg5309sup1.cif

Structure factors: contains datablock(s) I. DOI: 10.1107/S1600536813010830/hg5309Isup2.hkl

Supplementary material file. DOI: 10.1107/S1600536813010830/hg5309Isup3.cml

Additional supplementary materials:  crystallographic information; 3D view; checkCIF report

## Figures and Tables

**Table 1:** Hydrogen-bond geometry (Å, °)

*D*—H⋯*A*	*D*—H	H⋯*A*	*D*⋯*A*	*D*—H⋯*A*
C13—H13⋯O1^i^	0.96	2.37	3.254 (4)	153

Symmetry code: (i) 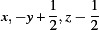.

**Fig. 1.**
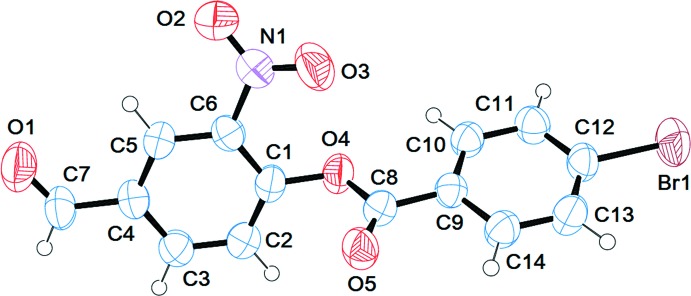
Molecular conformation and atom numbering scheme for the title compound with displacement ellipsoids drawn at the 50% probability level. H atoms are shown as spheres of arbitrary radius.

**Fig. 2.**
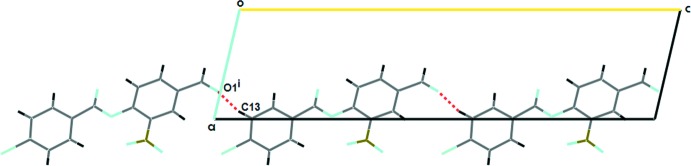
Part of the crystal structure of (I), showing the formation of chains which running along [001]. Symmetry code: (i) x,-y+1/2,+z-1/2.

## supplementary crystallographic information

### Comment

Esters containing aromatic nitro-substituted rings can be used as precursors for the preparation of compounds with potential analgesic and anti-inflammatory properties (Jefford & Zaslona, 1985). Indeed, many pharmaceuticals come from a variety of nitroaromatic compounds. Acetaminophen for example, a widely used drug, is sinthesized from p-nitrophenol (Bhattacharya *et al.*, 2006). Other nitro-aromatic esters show marked inhibitory activity against ischemic-induced electrocardiographic changes (Benedini *et al.*, 1995). In order to complement the structural information about nitroaryl compounds the title ester, 4-formyl-2-nitrophenyl 4-bromo benzoate, (I), was synthesized. The molecular structure of (I) is shown in Fig. 1. Bond lengths and bond angles in (I), show marked similarity with other aryl benzoates reported in the literature such as 4-methylphenyl 4-bromobenzoate (MPBrB) (Moreno-Fuquen *et al.*, 2011), 4-nitrophenyl 4-bromobenzoate (NPBrB) (Moreno-Fuquen, 2011) and 2,4,6-trinitrophenyl-4-chlorobenzoate (TNPClB) (Moreno-Fuquen *et al.*, 2013). However, it was noticed in (I) that the bond length O4-C1 in the ester moety, is shortened if it is compared with analogous distances in systems like MPBrB, NPBrB and the majority of similar aromatic esters. This behavior is comparable with those ones well described for trinitro-phenyl benzoates, such as TNPClB, as a consequence of resonance effects over the structure, prominently caused for ortho-nitro-substitution. The benzene rings of (I) form a dihedral angle of 62.90 (7)°, a value close to the value presented in TNPClB and NPBrB systems [63.46 (5)° and 64.98 (10)°] respectively. The ester group is twisted away from the nitro-substituted and bromo-substituted benzene rings by 71.67 (7)° and 8.78 (15)° respectively. The nitro group forms a dihedral angle with the benzene ring to which it is attached of 7.77 (16)°.

The crystal packing shows no classical hydrogen bonds and it is stabilized by weak C-H···O intermolecular interactions, forming C(12) chains along [001] (see Fig. 2; Etter, 1990). The C13 atom of the benzoic ring at (x,y,z) acts as hydrogen-bond donors to O1 atom at (x,-y+1/2,+z-1/2) (see Table 1; Nardelli, 1995).

Recent theoretical calculations show that halogen···halogen interactions are controlled by electrostatic forces and they display directional character (Awwadi *et al.*, 2006). In the title structure, halogen···halogen interactions [Br···Br = 3.523 (3) Å] within the chains stabilized by C—H···O interactions are observed. This Br···Br distance is much shorter than the sum of the van der Waals radii (3.70 Å) (Bondi, 1964). These interactions can be considered type I with trans geometry (Hathwar *et al.*, 2010).

### Experimental

The reagents and solvents for the synthesis were obtained from the Aldrich Chemical Co., and were used without additional purification. In a 100 ml round bottom flask 4-hydroxy-3-nitrobenzaldehyde (0.571 mmol, 0.20 g) and 4-bromobenzoyl chloride in equimolar amounts, were dissolved in 20 mL of acetonitrile. Also a few drops of pyridine were added. Then the mixture was left to reflux in constant stirring for about two hours. A colourless solid was obtained after leaving the solvent to evaporate. IR spectra were recorded on a FT-IR SHIMADZU IR-Affinity-1 spectrophotometer. Colourless crystals; m.p 422 (1)K. IR (KBr) 3101.90 cm^-1^, 3072.12 cm^-1^ (aromatic C-H); 1741.61 cm^-^1 (benzaldehyde C=O); 1710.5 cm^-1^ (ester C=O), 1228.04 cm^-1^ (ester C-O); 1533.35 cm^-1^, 1339.51 cm^-1^ (nitro –NO_2_); 1072.81 cm^-1^ (C=C); 742.43 cm^-1^(Br-C).

### Refinement

All the H-atoms attached to C atoms were positioned at geometrically idealized positions and treated as riding with C—H= 0.96 Å and *U*_iso_(H) = 1.2 *U*_eq_(C).

### Crystal data

**Table:**

C_14_H_8_BrNO_5_	*F*(000) = 696
*M**_r_* = 350.12	*D*_x_ = 1.731 Mg m^−^^3^
Monoclinic, *P*2_1_/*c*	Melting point: 422(1) K
Hall symbol: -P 2ybc	Mo *K*α radiation, λ = 0.71073 Å
*a* = 6.5308 (2) Å	Cell parameters from 3737 reflections
*b* = 8.2253 (2) Å	θ = 3.0–26.4°
*c* = 25.6860 (8) Å	µ = 3.08 mm^−^^1^
β = 103.1910 (9)°	*T* = 295 K
*V* = 1343.38 (7) Å^3^	Prism, colourless
*Z* = 4	0.38 × 0.16 × 0.12 mm

### Data collection

**Table:**

Nonius KappaCCD diffractometer	2746 independent reflections
Radiation source: fine-focus sealed tube	2095 reflections with *I* > 2σ(*I*)
Graphite monochromator	*R*_int_ = 0.086
CCD rotation images, thick slices scans	θ_max_ = 26.4°, θ_min_ = 3.0°
Absorption correction: multi-scan (*SADABS*; Sheldrick, 1996)	*h* = −8→6
*T*_min_ = 0.568, *T*_max_ = 0.687	*k* = −10→9
16446 measured reflections	*l* = −32→32

### Refinement

**Table:**

Refinement on *F*^2^	Primary atom site location: structure-invariant direct methods
Least-squares matrix: full	Secondary atom site location: difference Fourier map
*R*[*F*^2^ > 2σ(*F*^2^)] = 0.041	Hydrogen site location: inferred from neighbouring sites
*wR*(*F*^2^) = 0.113	H-atom parameters constrained
*S* = 1.03	*w* = 1/[σ^2^(*F*_o_^2^) + (0.0507*P*)^2^ + 0.4984*P*] where *P* = (*F*_o_^2^ + 2*F*_c_^2^)/3
2746 reflections	(Δ/σ)_max_ < 0.001
190 parameters	Δρ_max_ = 0.43 e Å^−^^3^
0 restraints	Δρ_min_ = −0.39 e Å^−^^3^

### Special details

**Table:**

Geometry. All esds (except the esd in the dihedral angle between two l.s. planes) are estimated using the full covariance matrix. The cell esds are taken into account individually in the estimation of esds in distances, angles and torsion angles; correlations between esds in cell parameters are only used when they are defined by crystal symmetry. An approximate (isotropic) treatment of cell esds is used for estimating esds involving l.s. planes.
Refinement. Refinement of *F*^2^ against ALL reflections. The weighted *R*-factor *wR* and goodness of fit *S* are based on *F*^2^, conventional *R*-factors *R* are based on *F*, with *F* set to zero for negative *F*^2^. The threshold expression of *F*^2^ > σ(*F*^2^) is used only for calculating *R*-factors(gt) *etc*. and is not relevant to the choice of reflections for refinement. *R*-factors based on *F*^2^ are statistically about twice as large as those based on *F*, and *R*- factors based on ALL data will be even larger.

### Fractional atomic coordinates and isotropic or equivalent isotropic displacement parameters (Å^2^)

**Table:**

	*x*	*y*	*z*	*U*_iso_*/*U*_eq_	
Br1	1.31938 (6)	0.42327 (5)	0.035355 (14)	0.08659 (19)	
O1	0.7475 (5)	0.4084 (3)	0.49383 (11)	0.0950 (8)	
O2	1.3030 (4)	0.2431 (3)	0.40032 (11)	0.0952 (8)	
N1	1.1928 (4)	0.2760 (3)	0.35705 (11)	0.0598 (6)	
C1	0.8918 (4)	0.4497 (3)	0.31011 (11)	0.0499 (6)	
C2	0.7088 (4)	0.5329 (3)	0.31062 (12)	0.0587 (7)	
H2	0.6359	0.5890	0.2790	0.070*	
C3	0.6341 (5)	0.5347 (3)	0.35660 (12)	0.0600 (7)	
H3	0.5051	0.5909	0.3563	0.072*	
C4	0.7415 (4)	0.4571 (3)	0.40263 (11)	0.0542 (6)	
C5	0.9260 (4)	0.3742 (3)	0.40244 (10)	0.0509 (6)	
H5	1.0040	0.3205	0.4339	0.061*	
C6	0.9988 (4)	0.3692 (3)	0.35613 (11)	0.0487 (6)	
O3	1.2303 (4)	0.2336 (3)	0.31487 (10)	0.0826 (7)	
O4	0.9767 (3)	0.4597 (2)	0.26543 (7)	0.0585 (5)	
O5	0.7279 (3)	0.2919 (2)	0.21998 (8)	0.0638 (5)	
C7	0.6609 (5)	0.4666 (4)	0.45221 (12)	0.0622 (7)	
H7	0.5332	0.5250	0.4518	0.075*	
C8	0.8811 (4)	0.3721 (3)	0.22136 (10)	0.0495 (6)	
C9	0.9929 (4)	0.3918 (3)	0.17757 (10)	0.0472 (6)	
C10	1.1847 (4)	0.4683 (3)	0.18437 (11)	0.0525 (6)	
H10	1.2502	0.5140	0.2185	0.063*	
C11	1.2847 (4)	0.4808 (3)	0.14219 (12)	0.0575 (7)	
H11	1.4190	0.5329	0.1463	0.069*	
C12	1.1860 (5)	0.4152 (3)	0.09351 (11)	0.0557 (7)	
C13	0.9933 (5)	0.3400 (4)	0.08568 (12)	0.0630 (7)	
H13	0.9283	0.2961	0.0512	0.076*	
C14	0.8966 (4)	0.3285 (3)	0.12789 (11)	0.0576 (7)	
H14	0.7617	0.2769	0.1231	0.069*	

### Atomic displacement parameters (Å^2^)

**Table:**

	*U*^11^	*U*^22^	*U*^33^	*U*^12^	*U*^13^	*U*^23^
Br1	0.0923 (3)	0.1169 (4)	0.0618 (3)	−0.00649 (19)	0.0410 (2)	0.00527 (18)
O1	0.112 (2)	0.121 (2)	0.0613 (17)	0.0260 (15)	0.0380 (15)	0.0109 (14)
O2	0.0753 (14)	0.133 (2)	0.0756 (17)	0.0367 (14)	0.0142 (13)	0.0138 (15)
N1	0.0586 (13)	0.0586 (13)	0.0649 (16)	0.0002 (10)	0.0197 (12)	−0.0011 (12)
C1	0.0572 (15)	0.0541 (14)	0.0397 (14)	−0.0125 (11)	0.0138 (11)	−0.0027 (11)
C2	0.0602 (16)	0.0662 (16)	0.0492 (16)	0.0020 (13)	0.0113 (13)	0.0072 (13)
C3	0.0592 (16)	0.0650 (17)	0.0578 (18)	0.0083 (13)	0.0171 (14)	0.0017 (14)
C4	0.0619 (16)	0.0555 (15)	0.0495 (16)	0.0002 (12)	0.0217 (13)	−0.0024 (12)
C5	0.0609 (16)	0.0520 (13)	0.0407 (14)	−0.0010 (12)	0.0138 (12)	0.0011 (11)
C6	0.0511 (14)	0.0451 (12)	0.0525 (15)	−0.0026 (11)	0.0168 (12)	−0.0029 (11)
O3	0.0878 (15)	0.0896 (15)	0.0811 (16)	0.0140 (12)	0.0415 (13)	−0.0103 (13)
O4	0.0624 (11)	0.0732 (12)	0.0424 (11)	−0.0174 (9)	0.0169 (9)	−0.0027 (9)
O5	0.0610 (11)	0.0761 (12)	0.0576 (12)	−0.0208 (10)	0.0202 (9)	−0.0045 (9)
C7	0.078 (2)	0.0668 (17)	0.0487 (17)	0.0074 (14)	0.0293 (15)	−0.0008 (14)
C8	0.0528 (15)	0.0511 (13)	0.0439 (14)	−0.0011 (12)	0.0096 (11)	0.0023 (11)
C9	0.0505 (14)	0.0475 (13)	0.0444 (14)	0.0002 (10)	0.0125 (11)	0.0026 (11)
C10	0.0539 (15)	0.0569 (14)	0.0460 (15)	−0.0046 (12)	0.0101 (12)	−0.0037 (12)
C11	0.0564 (15)	0.0607 (16)	0.0581 (18)	−0.0075 (13)	0.0184 (13)	0.0011 (13)
C12	0.0646 (16)	0.0621 (16)	0.0442 (15)	0.0054 (13)	0.0205 (13)	0.0077 (12)
C13	0.0703 (18)	0.0736 (18)	0.0439 (15)	−0.0067 (15)	0.0104 (13)	−0.0018 (13)
C14	0.0554 (15)	0.0690 (17)	0.0479 (16)	−0.0100 (13)	0.0105 (12)	−0.0014 (13)

### Geometric parameters (Å, º)

**Table:**

Br1—C12	1.894 (3)	C5—H5	0.9601
O1—C7	1.190 (4)	O4—C8	1.367 (3)
O2—N1	1.209 (3)	O5—C8	1.192 (3)
N1—O3	1.215 (3)	C7—H7	0.9599
N1—C6	1.476 (3)	C8—C9	1.483 (3)
C1—C2	1.380 (4)	C9—C10	1.377 (4)
C1—O4	1.386 (3)	C9—C14	1.389 (4)
C1—C6	1.395 (4)	C10—C11	1.391 (4)
C2—C3	1.377 (4)	C10—H10	0.9599
C2—H2	0.9599	C11—C12	1.380 (4)
C3—C4	1.385 (4)	C11—H11	0.9600
C3—H3	0.9600	C12—C13	1.375 (4)
C4—C5	1.385 (4)	C13—C14	1.377 (4)
C4—C7	1.487 (4)	C13—H13	0.9601
C5—C6	1.379 (3)	C14—H14	0.9600
			
O2—N1—O3	123.8 (3)	O1—C7—H7	116.4
O2—N1—C6	117.4 (2)	C4—C7—H7	119.5
O3—N1—C6	118.8 (2)	O5—C8—O4	122.6 (2)
C2—C1—O4	119.3 (2)	O5—C8—C9	126.4 (2)
C2—C1—C6	119.7 (3)	O4—C8—C9	111.1 (2)
O4—C1—C6	120.8 (2)	C10—C9—C14	119.7 (2)
C3—C2—C1	119.4 (3)	C10—C9—C8	123.0 (2)
C3—C2—H2	121.0	C14—C9—C8	117.2 (2)
C1—C2—H2	119.6	C9—C10—C11	120.7 (3)
C2—C3—C4	121.2 (3)	C9—C10—H10	119.8
C2—C3—H3	118.8	C11—C10—H10	119.5
C4—C3—H3	120.0	C12—C11—C10	118.1 (2)
C3—C4—C5	119.7 (3)	C12—C11—H11	120.0
C3—C4—C7	119.9 (3)	C10—C11—H11	121.8
C5—C4—C7	120.5 (3)	C13—C12—C11	122.1 (3)
C6—C5—C4	119.3 (2)	C13—C12—Br1	118.1 (2)
C6—C5—H5	119.5	C11—C12—Br1	119.8 (2)
C4—C5—H5	121.2	C12—C13—C14	119.0 (3)
C5—C6—C1	120.8 (2)	C12—C13—H13	120.1
C5—C6—N1	117.8 (2)	C14—C13—H13	120.9
C1—C6—N1	121.5 (2)	C13—C14—C9	120.3 (3)
C8—O4—C1	117.44 (19)	C13—C14—H14	120.1
O1—C7—C4	124.0 (3)	C9—C14—H14	119.6

### Hydrogen-bond geometry (Å, º)

**Table:**

*D*—H···*A*	*D*—H	H···*A*	*D*···*A*	*D*—H···*A*
C13—H13···O1^i^	0.96	2.37	3.254 (4)	153

Symmetry code: (i) *x*, −*y*+1/2, *z*−1/2.
